# Natural history of *Sudan ebolavirus* infection in rhesus and cynomolgus macaques

**DOI:** 10.1080/22221751.2022.2086072

**Published:** 2022-06-14

**Authors:** Courtney Woolsey, Alyssa C. Fears, Viktoriya Borisevich, Krystle N. Agans, Natalie S. Dobias, Abhishek N. Prasad, Daniel J. Deer, Joan B. Geisbert, Karla A. Fenton, Thomas W. Geisbert, Robert W. Cross

**Affiliations:** a Department of Microbiology and Immunology, University of Texas Medical Branch, Galveston, TX, USA; b Galveston National Laboratory, University of Texas Medical Branch, Galveston, TX, USA

**Keywords:** *Sudan ebolavirus*, Sudan virus, filovirus, Ebola virus, natural history, nonhuman primates, macaques, animal models

## Abstract

Due to its high mortality rate and continued re-emergence, Ebolavirus disease (EVD) continues to pose a serious threat to global health. A group of viruses within the genus *Ebolavirus* causes this severe hemorrhagic disease in humans: Ebola virus (EBOV; species *Zaire ebolavirus*), Sudan virus (SUDV; species *Sudan ebolavirus*), Bundibugyo virus, and Taï Forest virus. EBOV and SUDV are associated with the highest case fatality rates. While the host response to EBOV has been comprehensively examined, limited data exists for SUDV infection. For medical countermeasure testing, well-characterized SUDV nonhuman primate (NHP) models are thus needed. Here, we describe a natural history study in which rhesus (*N* = 11) and cynomolgus macaques (*N* = 14) were intramuscularly exposed to a 1000 plaque-forming unit dose of SUDV (Gulu variant). Time-course analyses of various hematological, pathological, serological, coagulation, and transcriptomic findings are reported. SUDV infection was uniformly lethal in cynomolgus macaques (100% mortality), whereas a single rhesus macaque subject (91% mortality) survived to the study endpoint (median time-to-death of ∼8.0 and ∼8.5 days in cynomolgus and rhesus macaques, respectively). Infected macaques exhibited hallmark features of human EVD. The early stage was typified by viremia, granulocytosis, lymphopenia, albuminemia, thrombocytopenia, and decreased expression of HLA-class transcripts. At mid-to-late disease, animals developed fever and petechial rashes, and expressed high levels of pro-inflammatory mediators, pro-thrombotic factors, and markers indicative of liver and kidney injury. End-stage disease was characterized by shock and multi-organ failure. In summary, macaques recapitulate human SUDV disease, supporting these models for use in the development of vaccines and therapeutics.

## Introduction

The *Ebolavirus* genus (family *Filoviridae*) is comprised of six recognized species: *Reston ebolavirus*, *Bombali ebolavirus*, *Zaire ebolavirus*, *Sudan ebolavirus*, *Bundibugyo ebolavirus*, and *Taï Forest ebolavirus* [1]. African fruit bats are the purported natural reservoirs of these filamentous, enveloped, non-segmented, negative-sense RNA viruses [2]. Reston virus (RESTV; species *Reston ebolavirus*) causes disease in nonhuman primates and pigs, but no cases have been reported in people [3–5]. In 2018, Bombali virus (BOMV; species *Bombali ebolavirus*) was discovered in bats in Sierra Leone [2], but it is still unclear whether the virus is pathogenic in humans or animals [6]. The other filovirus species comprise a group of viruses that are important human pathogens with case-fatality rates ranging from 50% to 90% for Ebola virus (EBOV; species *Zaire ebolavirus*), ∼ 55% for Sudan virus (SUDV; species *Sudan ebolavirus*), and ∼25-50% for Bundibugyo virus (BDBV; species *Bundibugyo ebolavirus*) [1, 7–9]. Taï Forest virus (TAFV; species *Taï Forest ebolavirus*) was the culprit of a single non-fatal case of Ebola virus disease (EVD) [1, 10].

SUDV and EBOV have been responsible for most cases of EVD and were the first ebolaviruses discovered. These viruses appeared to emerge simultaneously in the summer of 1976 in the Sudanese towns of Nzara and Maridi and Yambuku, Democratic Republic of Congo (formerly Zaire), respectively [7], and ostensibly spread independently to people in each of the affected areas. Emerging filovirus species causing outbreaks in Central and West Africa are hypothesized to coincide with the proportion of seropositivity to each filovirus species in fruit bats or other potential reservoirs. For example, filoviruses causing outbreaks in West and Central Africa between 2005 and 2012 shifted from Zaire ebolaviruses to Sudan and Bundibugyo ebolaviruses, concurrent with a change in the serologically dominant virus species in bats [11]. Further investigation of SUDV in animal and human hosts is clearly needed given its historic serodominance in bats, high lethality, and high potential for future spillover into human populations.

In humans and preclinical animal models, the host response to EBOV is well-characterized, whereas limited data is available for SUDV infection. In rodents (mice, guinea pigs, and hamsters), adaptation of filoviruses is necessary to cause lethal disease in immunocompetent hosts [12,13]. Typically, rodent models do not exhibit the disordered coagulopathy seen in human EVD cases, e.g. pro-thrombotic changes or extensive fibrin deposition. Ferrets are susceptible to wild-type SUDV and exhibit some of the coagulopathies associated with human infection [14,15], but few immunological reagents are available to study host responses. Nonhuman primates (NHPs), particularly macaque species, are considered the “gold standard” animal model for filovirus infection as they recapitulate most human manifestations of EVD including the development of disseminated intravascular coagulation and a maculopapular rash [16]. Macaques are highly susceptible to SUDV without the need for virus adaptation [17], and ample immunological resources are available to study the immune response.

To date, only an aerosol model of SUDV infection has been characterized in an NHP model [17]. Here, we describe a detailed natural history study of cynomolgus macaques (CM) and rhesus macaques (RM) exposed to 1000 plaque-forming units of SUDV via the intramuscular (i.m.) route. The i.m. model is important as it represents the most lethal route of infection (e.g. needlestick) and is the standard for evaluating medical countermeasures as well as serving as a comparison for other filovirus models (i.e. there are limited mucosal challenge data available). Virology and various hematological, pathological, serological, coagulation, and transcriptomic findings are reported. Our results shed light on the pathophysiology of SUDV infection and highlight the utility of NHPs for testing medical countermeasures against this deadly pathogen.

## Materials and methods

### Ethics statement

Animal studies were conducted in compliance with the Animal Welfare Act and other federal statutes and regulations relating to animal experimentation. Details are provided in the Supplemental Methods.

### Challenge virus

The SUDV seed stock (Gulu variant) originates from the serum of a fatal patient (35-year-old male; isolate 200011676) during the 2000–2001 Uganda outbreak (RefSeq #NC_006432). Details are provided in the Supplemental Methods.

### Animal challenge

Fourteen cynomolgus (*Macaca fascicularis*) [18–20] and eleven rhesus (*Macaca mulatta*) [21–24] macaques of Chinese origin (PreLabs, Worldwide Primates) that served as virus positive controls from 17 studies at the Galveston National Laboratory (GNL) were employed for this project. Results from the remaining seven cynomolgus and three rhesus macaques have not been published. Details are provided in the Supplemental Methods.

### Blood collection

Blood was collected by venipuncture into EDTA and serum tubes throughout the course of the study. Details are provided in the Supplemental Methods.

### Hematology and clinical chemistry

Blood samples were analyzed using a laser-based hematologic analyzer and serum samples were tested using a Piccolo point-of-care analyzer and Biochemistry Panel Plus analyzer discs (Abaxis). Details are provided in the Supplemental Methods.

### Viral load determination

SUDV viral loads were determined using RT-qPCR and standard plaque assays. Details are provided in the Supplemental Methods.

### Transcriptomics

Targeted transcriptomics was performed on macaque whole blood as previously described [25]. Immune cell profiling was accomplished via CIBERSORT web-based deconvolution software [26] using the LM22 signature matrix file. The 6-way Venn diagram was created with InteractiVenn [27]. Details are provided in the Supplemental Methods.

### Bead-based multiplex assays

Analytes were measured by flow cytometry using Biolegend LegendPlex™ assays and a FACS Canto-II cytometer (Becton Dickson) and plotted using the ggplot2 (v3.3.5) [28], ggbreak (v0.0.8) [29], viridis (v0.6.2) [30], and rstatix (v0.7.0) packages. Details are provided in the Supplemental Methods.

### Histopathology and immunohistochemistry

Tissue samples of all major organs were harvested in 10% neutral buffered formalin for histopathologic and immunohistochemical (IHC) examination. Slides were reviewed by a board-certified veterinary pathologist. Details are provided in the Supplemental Methods.

### Statistical analysis

Statistical comparisons were carried out in GraphPad Prism. Details are provided in the Supplemental Methods.

## Results

**Figure 1.**
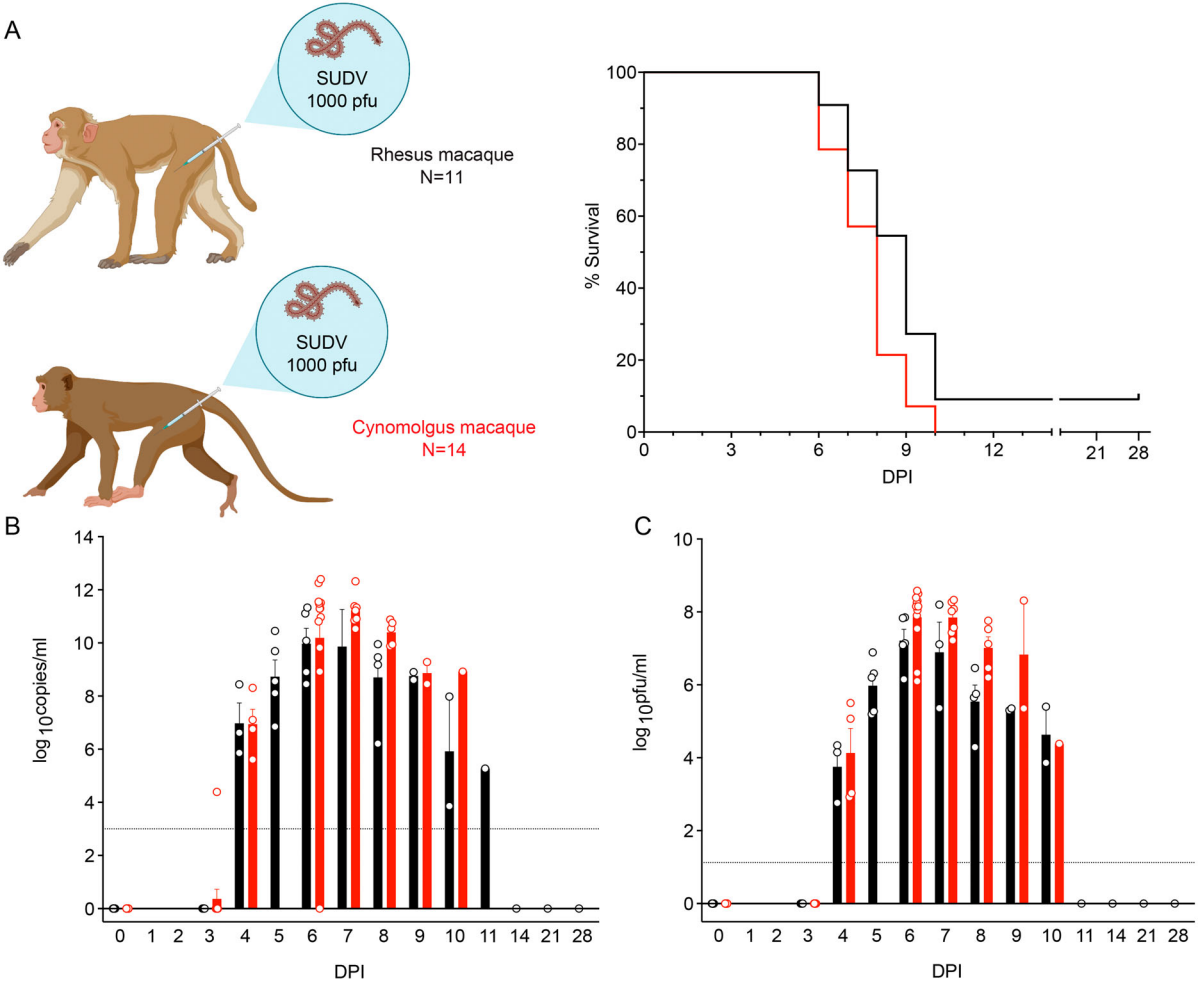
Survival and viral loads of SUDV-infected macaques. (A) Kaplan-Meier survival curves of cynomolgus macaques (red; *N* = 14) and rhesus macaques (black; *N* = 11) i.m. exposed to 1000 PFU of SUDV-Gulu. Macaque icons were created with BioRender (https://biorender.com/). (B) Viral loads were measured by RT-qPCR in whole blood and reported as log10 copies/ml at the denoted time points. The limit of detection for this assay is 1000 copies/ml (indicated by a dotted horizontal line). (C) Plasma viremia was measured by standard plaque assay at the denoted time points and reported as log10 PFU/ml. The limit of detection for this assay is 25 PFU/ml (indicated by a dotted horizontal line). For (B) and (C), each bar represents the average titre ± SEM for each cohort. Individual subjects are represented by circles. Abbreviations: SUDV, Sudan virus; i.m., intramuscular; DPI, days post infection; PFU, plaque-forming units.

Eleven rhesus macaques (RM) and fourteen cynomolgus macaques (CM) were i.m. challenged with a 1000 PFU target dose of SUDV-Gulu. The median time-to-death (TTD) for CM and RM was ∼8.0 (mean TTD 7.64 ± 1.72) and ∼8.5 days (mean TTD 8.3 ± 1.27), respectively. Disease was uniformly lethal in CM. A single RM subject survived to the 28-day post infection (DPI) study endpoint, corresponding to a 91% mortality rate within this group (Figure 1(A)). No statistically significant difference in survival was noted between the two species (log-rank test). Viral loads were assessed by performing RT-qPCR amplification and conventional plaque assays on whole blood and plasma samples, respectively. Viral RNA (vRNA) (Figure 1(B)) and infectious SUDV particles (Figure 1(C)) were first detected in both species at ∼ 4 DPI. Peak vRNA titres ranged from 7.2 to 12.4 log_10_ copies/ml, whereas infectious titres ranged from 5.2 to 8.6 log_10_ PFU/ml. CM compared to RM tended to have higher titres at 6–8 DPI, although no statistically significant difference was noted between the two cohorts for this parameter at any time point (Mann–Whitney *t*-tests). The single rhesus survivor had a peak titre of 5.1 log_10_ PFU/ml at 5 DPI, but no infectious virus was detected in this subject by 11 DPI.

**Figure 2.**
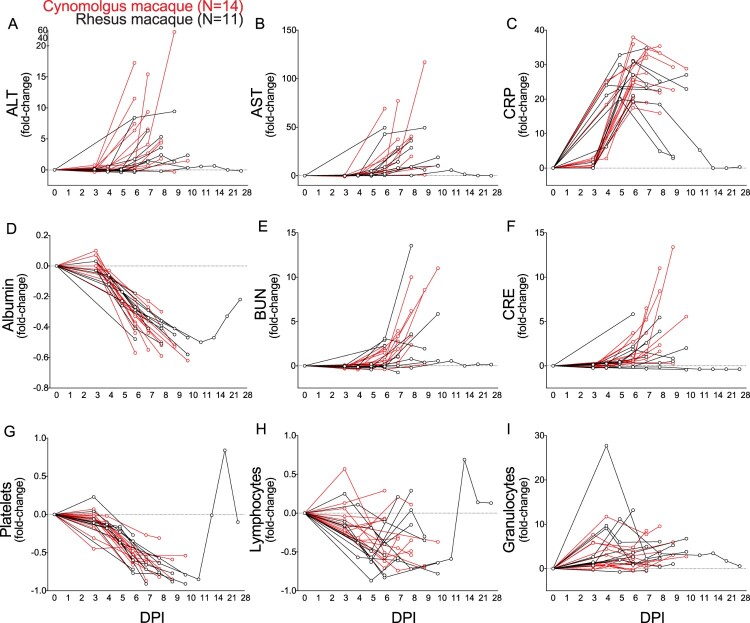
Notable clinical findings in SUDV-exposed macaques. (A-I) Each line represents repeated sampling of each individual SUDV-infected cynomolgus macaque (red; *N* = 14) or rhesus macaque (black; *N *= 11). (A-F) Fold-change clinical chemistry changes in the serum of infected cynomolgus and rhesus macaques at the denoted time points. (G-I) Fold-change hematological changes in the whole blood of infected cynomolgus and rhesus macaques at the denoted time points. Sampling timepoints for each subject are represented by circles. Abbreviations: SUDV, Sudan virus; alanine aminotransferase (ALT), aspartate aminotransferase (AST), C-reactive protein (CRP), blood urea nitrogen (BUN), creatine (CRE); DPI, days post infection.

SUDV-exposed macaques displayed hallmark features of human EVD (Figure 2, Tables S1 and S2). Early common clinical signs (3–4 DPI) included decreased food consumption, fever, lymphopenia, monocytosis, and granulocytosis. At mid-to-late disease (5–6 DPI), RM and CM exhibited anorexia, thrombocytopenia, hypoalbuminemia, hypoamylasemia, and a petechial rash. Liver enzymes (ALT, AST, ALP, GGT), markers indicative of kidney injury (BUN, CRE), and C-reactive protein (CRP; general inflammation marker) were elevated in all subjects at this stage. Preagonal disease (7-10 DPI) was characterized by weakness, lateral recumbency, and decreased body core temperature.

**Figure 3.**
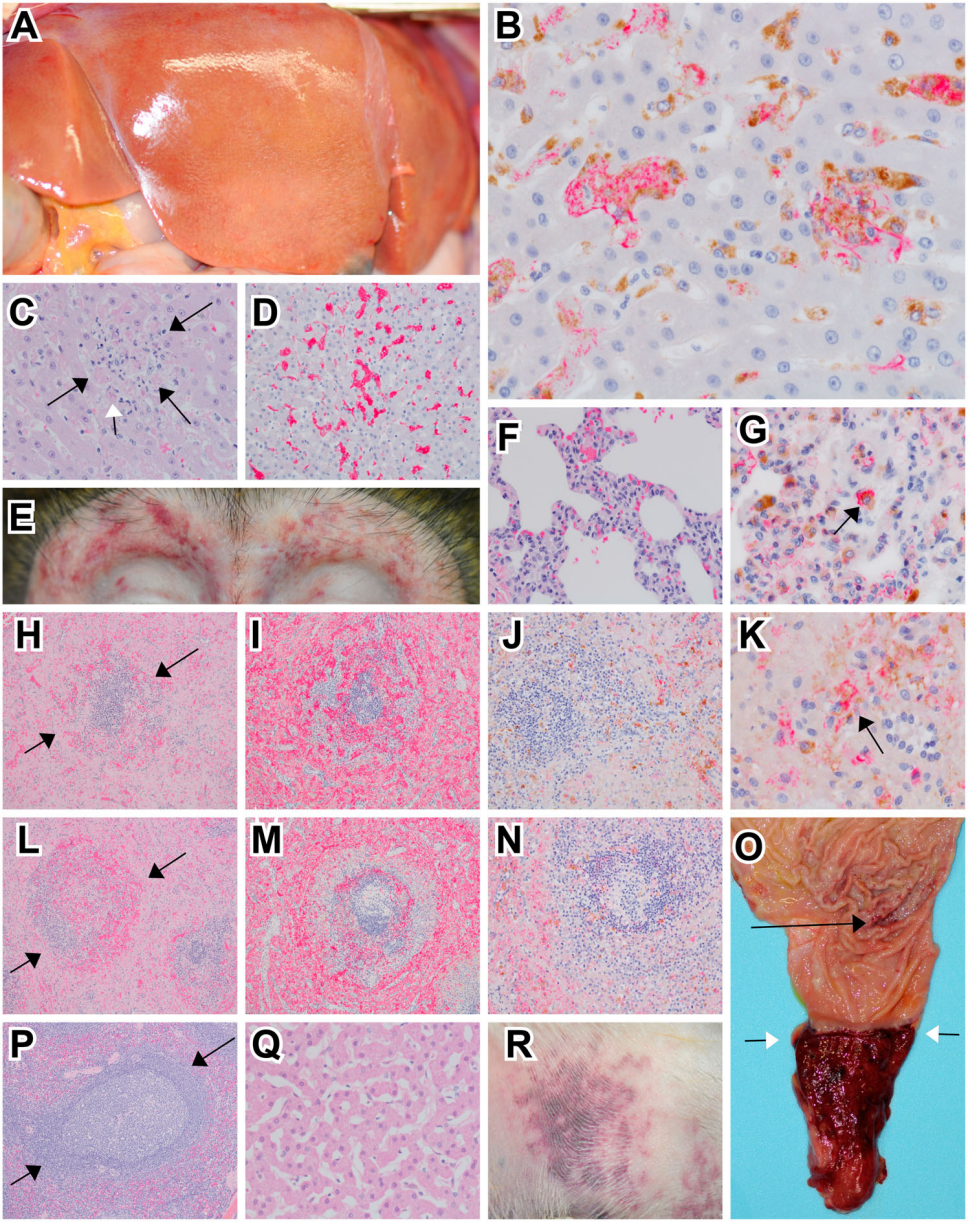
Representative gross and histologic lesions of SUDV-infected macaques. (A) Diffuse hepatic pallor indicative of hepatitis in cynomolgus macaque (RHES-1). (B) Immunohistochemistry (IHC) double labelling for macrophage (CD68-brown) and SUDV VP40 (red) in the liver of rhesus macaque (RHES-6) 40x, normal hepatic architecture was disrupted with expanded sinusoids that were occupied with clusters of macrophages infected with SUDV. (C) Hematoxylin and eosin (H&E) staining of the liver in rhesus macaque (RHES-6) 40x, normal hepatic architecture was disrupted with expanded sinusoids with mononuclear inflammation and eosinophilic cellular debris (black arrows), rarely eosinophilic intracytoplasmic inclusion bodies were noted (white arrow). (D) IHC for fibrin (red) in rhesus macaque (RHES-6) 20x, intra-sinusoidual fibrin deposition. (E) Bilateral periorbital petechial rash in rhesus macaque (RHES-2). (F) H&E staining of lung in cynomolgus macaque (CYNO-9) 40x, expansion of alveolar septa with mononuclear inflammatory cells and minimal extravasation of erythrocytes within alveoli. (G) IHC double labelling for macrophage (CD68-brown) and SUDV VP40 (red) in the lung of cynomolgus macaque (CYNO-9) 60x, alveolar macrophages were infected with SUDV (black arrow). (H) H&E staining of spleen in rhesus macaque (RHES-6) 10x, loss of normal white pulp architecture with extensive lymphocytolysis, hemorrhage and fibrin deposition (black arrows). (I) IHC labelling for fibrin (red) in the spleen of rhesus macaque (RHES-6) 10x, extensive fibrin deposition in red and white pulp. (J) IHC double labelling for macrophage (CD68-brown) and SUDV VP40 (red) in the spleen of rhesus macaque (RHES-6) 20x, macrophages infected with SUDV were sparsely present in the red and white pulp. (K) Higher magnification of IHC double labelling for macrophage (CD68-brown) and SUDV VP40 (red) in the spleen of rhesus macaque (RHES-6) 60x, macrophages infected with SUDV (black arrow). (L) H&E staining of spleen in cynomolgus macaque (CYNO-9) 10x, loss of normal white pulp architecture with extensive lymphocytolysis, hemorrhage and fibrin deposition (black arrows). (M) IHC labelling for fibrin (red) in the spleen of cynomolgus macaque (CYNO-9) 10x, extensive fibrin deposition in red and white pulp. (N) IHC double labelling for macrophage (CD68-brown) and SUDV VP40 (red) in the spleen of cynomolgus macaque (CYNO-9) 20x, macrophages infected with SUDV were sparsely present in the red and white pulp. (O) Mucosal surface of the pyloric region of the stomach and aboral duodenum in rhesus macaque (RHES-2), multifocal pinpoint ulcerations of the gastric mucosa (black arrow) and diffuse ulceration and hemorrhage of the aboral duodenum extending up to the pyloric sphincter (white arrows). (P) H&E staining of spleen in SUDV survivor rhesus macaque (RHES-7) 10x, no signification lesions. (Q) H&E staining of liver in SUDV survivor rhesus macaque (RHES-7) 10x, no signification lesions. (R) Petechial rash of the extremity in rhesus macaque (RHES-2).

Postmortem gross examination of animals at necropsy revealed one or more lesions consistent with EVD including petechial to ecchymotic rash, necrotizing hepatitis (characterized as hepatic pallor with reticulation), splenomegaly, lymphadenitis, hemorrhagic interstitial pneumonia, and gastrointestinal ulceration (Figure 3(A, E, R, O)).

Microscopically, tissue sections were consistent with EVD induced by SUDV infection. Predominant microscopic findings consisted of mixed inflammatory infiltrates largely composed of mononuclear cells that were IHC positive for SUDV antigen and widespread throughout multiple organs (Figure 3(B, G, J, K)). Inflammation was often accompanied with hemorrhage fibrin deposition and necrosis. Prominent fibrin deposition was noted particularly in highly vascularized organs such as the spleen and liver (Figure 3(D, I, M)). Conversely, the survivor lacked inflammatory findings, fibrin deposition, and immunolabeling for SUDV antigen (Figure 3(P, Q)).

Serology indicated that only the sole rhesus survivor (RHES-7) formed SUDV-specific antibodies (Table S2). In this subject, a low IgM titre (1:100) was detected by 8 DPI that subsided during the convalescent stage conjointly with the detection of moderate-to-high titres of IgG (1:800–1:3200) (Table S2).

**Figure 4.**
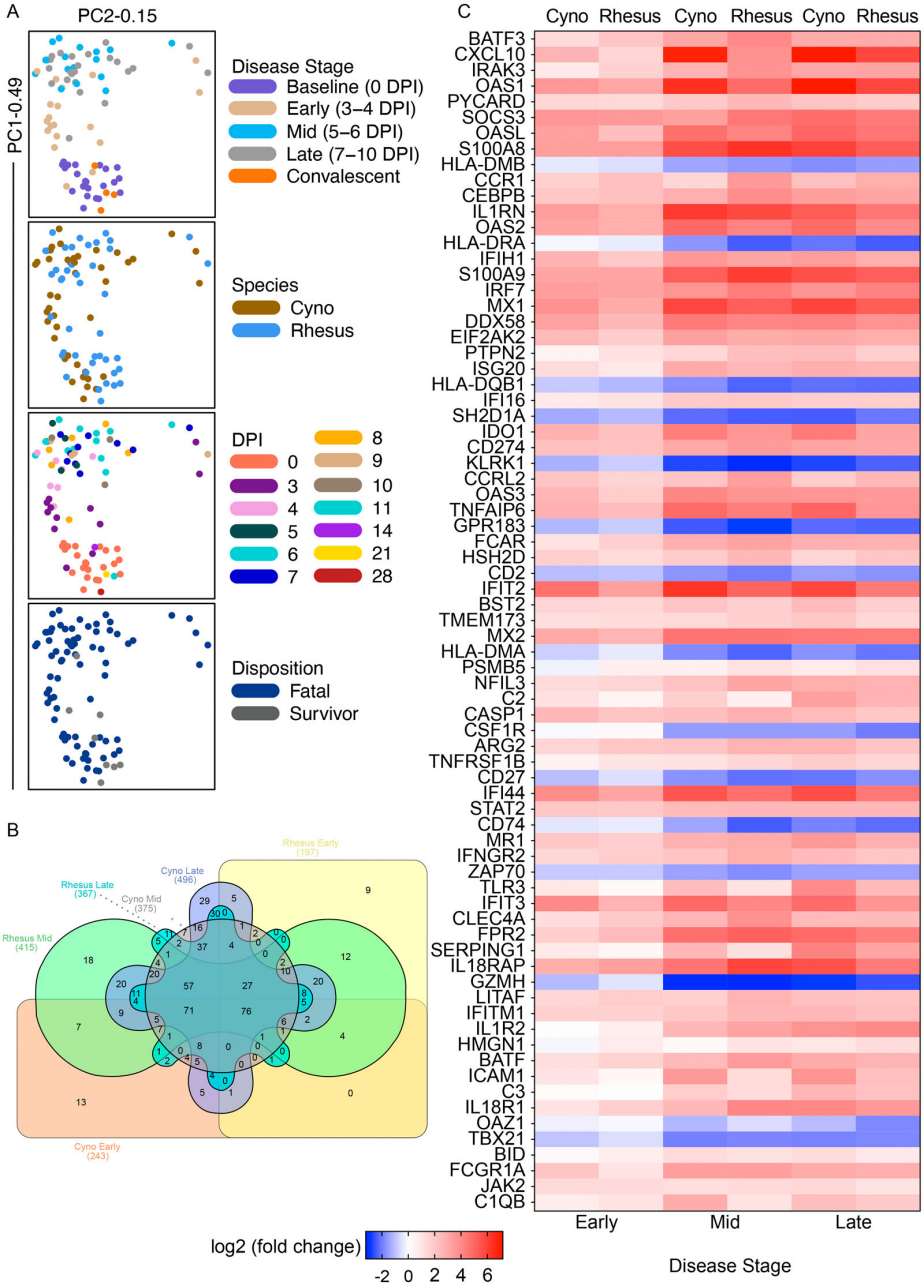
Transcriptional changes in SUDV-infected macaques. (A) Shown are principal component (PC) analyses of all normalized transcripts from cynomolgus macaques (red; *N* = 14) and rhesus macaques (black; *N* = 11) on the day of challenge (0 DPI), and at early (3–4 DPI), mid (5–6 DPI), late disease (7–10 DPI), and convalescence (14, 21, 28 DPI). Individual samples were filtered by disease stage (baseline, early, mid, late, convalescent), species (cyno, rhesus), DPI, or disposition (fatal, survivor). (B) 6-way Venn diagram depicting overlapping differentially expressed transcripts in each species at each disease stage. (C) Heatmap depicting the most differentially expressed transcripts in SUDV-infected macaques at each disease stage (multiple hypothesis Benjamini-Hochberg false discovery rate (FDR) corrected *p*-value less than 0.05). Red indicates upregulated transcripts; blue indicates downregulated transcripts; white indicates no change from baseline. Abbreviations: PC1 (principal component 1); PC2 (principal component 2); DPI, days post infection; cyno, cynomolgus macaque; rhesus, rhesus macaque; SUDV, Sudan virus.

To further characterize the immune response to SUDV infection, we performed targeted transcriptomics on whole blood RNA samples from RM (*N* = 10) or CM (*N* = 12) at baseline (pre-challenge), early (3–4 DPI), mid (5–6 DPI), and late (7–10 DPI) disease. One RM and two CM subjects were excluded from this analysis due to insufficient sample availability. Dimensional reduction via principal component analysis (PCA) showed most transcriptional changes occurred at the mid- and late-disease stages (Figure 4(A)). For both species, transcripts were mostly upregulated versus downregulated throughout the course of disease (Figure S1A). The survivor convalescent samples clustered with baseline RM and CM samples, indicating resolution of disease in this animal and a return to baseline mRNA levels by 11 DPI. Limited species-specific transcriptional differences were noted among SUDV-infected macaques, which comports with our survival, viremia, and clinical data (Figure 4(B,C)). A majority of differentially expressed (DE) transcripts (adjusted *p*-values < 0.05) was shared between different species and across each disease stage. Analysis of global changes showed repressed transcripts were associated with antigen processing and presentation (*HLA-DMB*, *HLA-DRA*, *HLA-DQB1*, *HLA-DMA*, *CD74*) and lymphocyte activation (*SH2D1A*, *KLRK1*, *CD2*, *ZAP70*, *GZMH*, *TBX21*, *CD27*, *GPR183*) (Figure 4(C)). The topmost upregulated molecules were involved in inflammation (e.g. *CXCL10*, *IL1RN*, *IL18RAP*, *TNFAIP6*), viral RNA sensing (e.g. *OAS1*, *OASL*, *MX1*, *DDX58*, *IFIH1*), interferon induction (e.g. *IFIT2*, *IFI44*), and monocyte/granulocyte accumulation and chemotaxis (e.g. *S100A8*, *S100A9*, *CCR1*).

**Figure 5.**
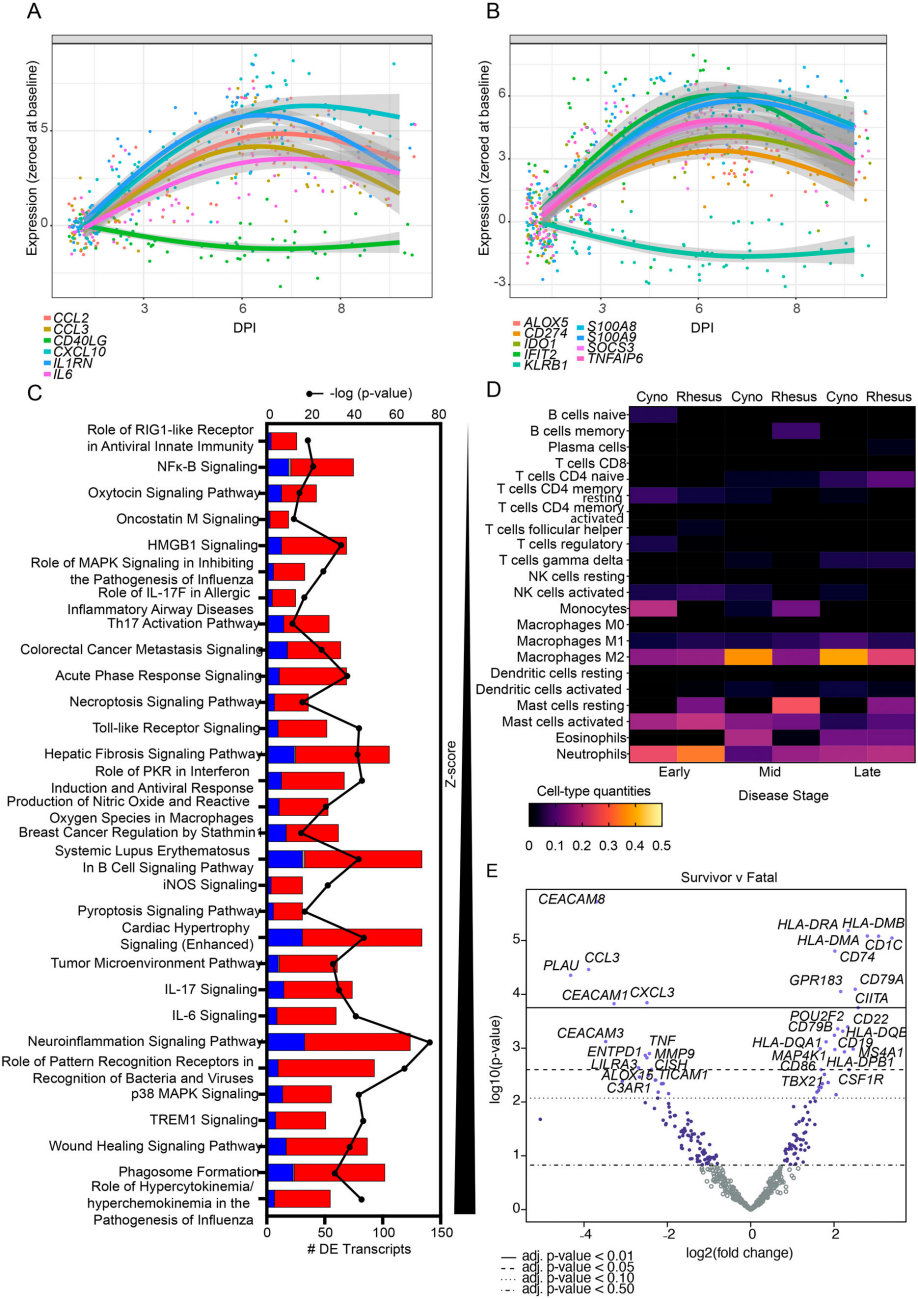
Biomarker discovery, pathway analysis, and immune cell type profiling of macaques exposed to SUDV. Trend plots depicting (A) historical or (B) novel transcriptional biomarkers for SUDV-infected fatal macaque subjects. (C) Pathway enrichment of differentially expressed transcripts in fatal SUDV-infected macaques sorted by Z-score and -log (*p*-value). Red indicates upregulated transcripts; blue indicates downregulated transcripts; grey indicates no change. (D) Respective cell-type quantities for SUDV-infected macaques at each disease stage. (E) Volcano plot displaying overall -log10(*p*-values) and log2 fold changes for each mRNA target in the single rhesus survivor versus fatal SUDV-infected macaques. Horizontal lines within the plot indicate adjusted *p*-value thresholds. Targets highlighted in blue indicate those differentially expressed in the survivor versus fatal group. A multiple hypothesis Benjamini-Hochberg false discovery rate (FDR) corrected *p*-value less than 0.05 was deemed significant for transcripts unless otherwise stated. Abbreviations: DPI, days post infection; cyno, cynomolgus macaque; rhesus, rhesus macaque; SUDV, Sudan virus.

To validate and demonstrate that systemic signatures in our NHP models match those seen in human SUDV disease, we compared previously reported biomarkers [31,32] to those identified using targeted transcriptomics in this study. The NHP models harmonized well with human infection with detection of biomarkers identified during SUDV outbreaks as early as 3 DPI. These biomarkers include MCP-1 (*CCL2*), M-CSF (*CCL3*), IP-10 (*CXCL10*), IL-1RA (*IL1RN*), and IL-6 (*IL6*) (Figure 5(A)). Consistent with human cases, expression of sCD40L (*CD40LG*) (sCD40-related transcript) was repressed in infected NHPs. To augment the current array of prognostic indicators, we identified a panel of novel biomarkers (*ALOX5*, *CD274*, *IDO1*, *IFIT2*, *KLRB1*, *S100A8*, *S100A9*, *SOCS3*, *TNFAIP6*) that were consistently upregulated (or downregulated in the case of *KLRB1*) throughout the course of disease beginning 3–4 DPI (Figure 5(B)).

**Figure 6.**
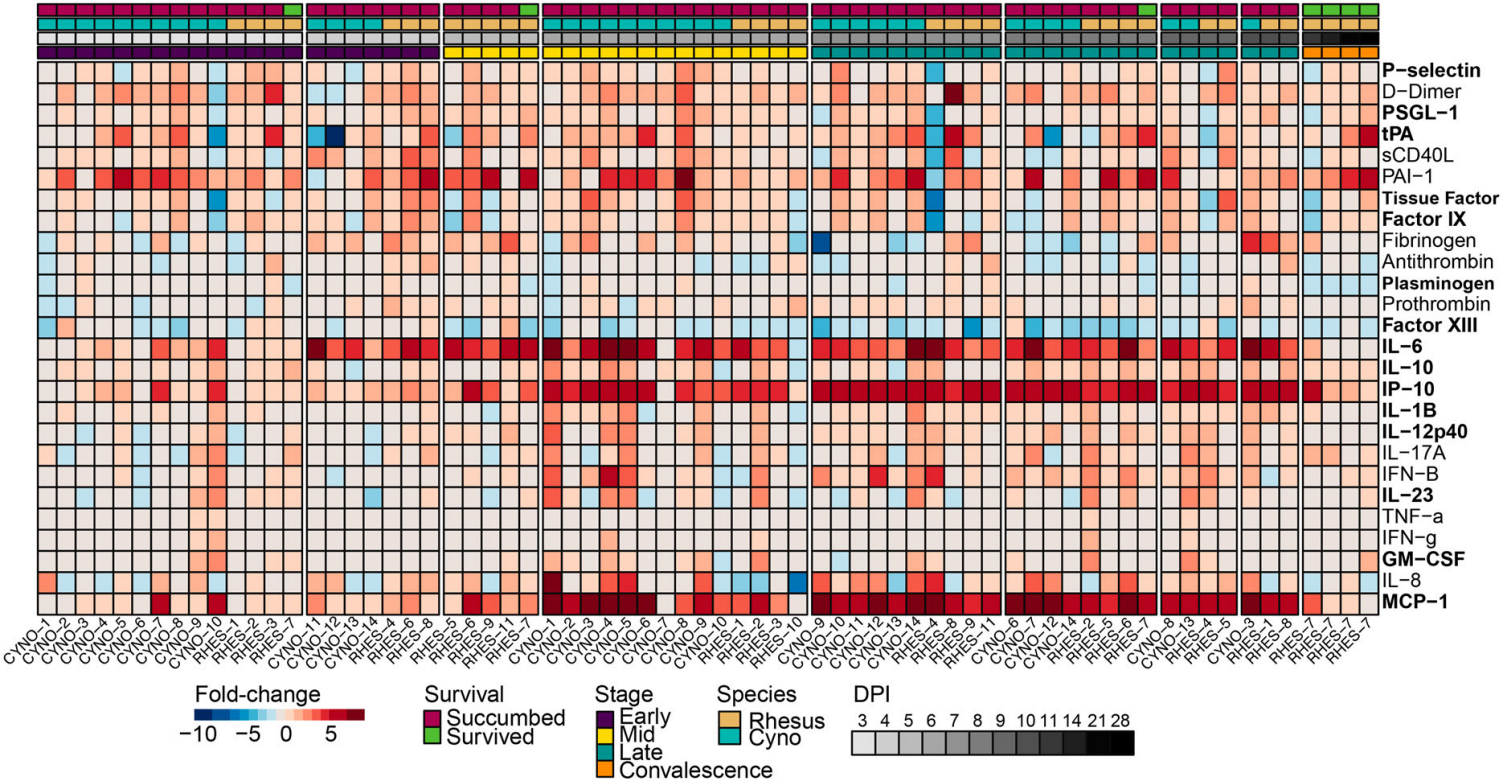
Expression of pro-inflammatory mediators and coagulation markers in the plasma of SUDV-infected macaques. Heatmap depicting expression fold-change values for individual cynomolgus or rhesus macaques with respect to a pre-challenge baseline. Red indicates increased expression; blue indicates decreased expression; white indicates no change in expression. Bolded text indicates a statistically significant analyte by ANOVA (*p* < 0.05). Abbreviations: DPI, days post infection; cyno, cynomolgus macaque; rhesus, rhesus macaque.

Functional enrichment of transcripts was executed to determine canonical signalling pathways and upstream regulators associated with SUDV infection. This analysis supported activation of pathways involved in cytokine/chemokine signalling, viral sensing, and antiviral immunity (Figure 5(C)). To capture shifts in circulating cell populations, we performed digital cell quantitation via transcriptional profiling (Figure 5(D)). SUDV infection of RM and CM was associated with predicted recruitment of monocyte, M2 macrophages, and various granulocyte populations (mast cells, eosinophils, and neutrophils), in accordance with our hematology results. Disease and function terms predominantly mapped to terms involved in myeloid cell phagocytosis (Figure S1B). The topmost upstream regulators included LPS, poly IC-RNA, TNF, IFNG, and IL1B, factors that have been previously identified for other ebolaviruses (Figure S1C) [25]. A pharmacological inhibitor of p38 mitogen-activated protein kinase (MAPK), SB203580 [33], was conversely predicted to significantly decrease. Congruent with our serum biochemistry results, predicted tox functions included terms associated with liver and kidney injury, e.g. “apoptosis of kidney cells,” “increased levels of ALT,” “increased levels of BUN,” and “acute renal failure” (Figure S1D). These transcriptional changes were evident by early disease.

To identify factors involved in natural defence against SUDV infection, we compared overall transcriptional changes in the single rhesus survivor (RHES-7) versus fatal animals. As opposed to fatal subjects, numerous antigen presentation molecules were expressed in the survivor (*HLA-DRA*, *HLA-DMB*, *HLA-DMA*, *CD1C*, *CIITA*) along with markers associated with activation of adaptive immunity (*CD74*, *GPR183*, *CD79A*, *CD79B*, *TBX21*) (Figure 5(E)). In contrast, decreased expression of transcripts involved in cell adhesion (*CEACAM8*, *CEACAM1*, *CEACAM3*) and migration (*CCL3*, *CXCL3*) were noted in the survivor, as well as plasminogen activator urokinase (*PLAU*).

Lastly, we measured protein levels of plasma-derived inflammatory mediator and thrombosis analytes. Like EBOV, SUDV disease was associated with elevated IL-6, IP-10, and MCP-1 concentrations (Figure 6, Figure S2) [31,32]. Of the 15 analytes that had a statistically significant association, 4 were cytokines or chemokines, and 3 of those – IL-10, IP-10, and RANTES – were associated with an age-dependent survival outcome. tPA levels were also increased, whereas plasminogen and Factor XIII were decreased, possibly due to consumptive coagulopathy.

**Figure 7.**
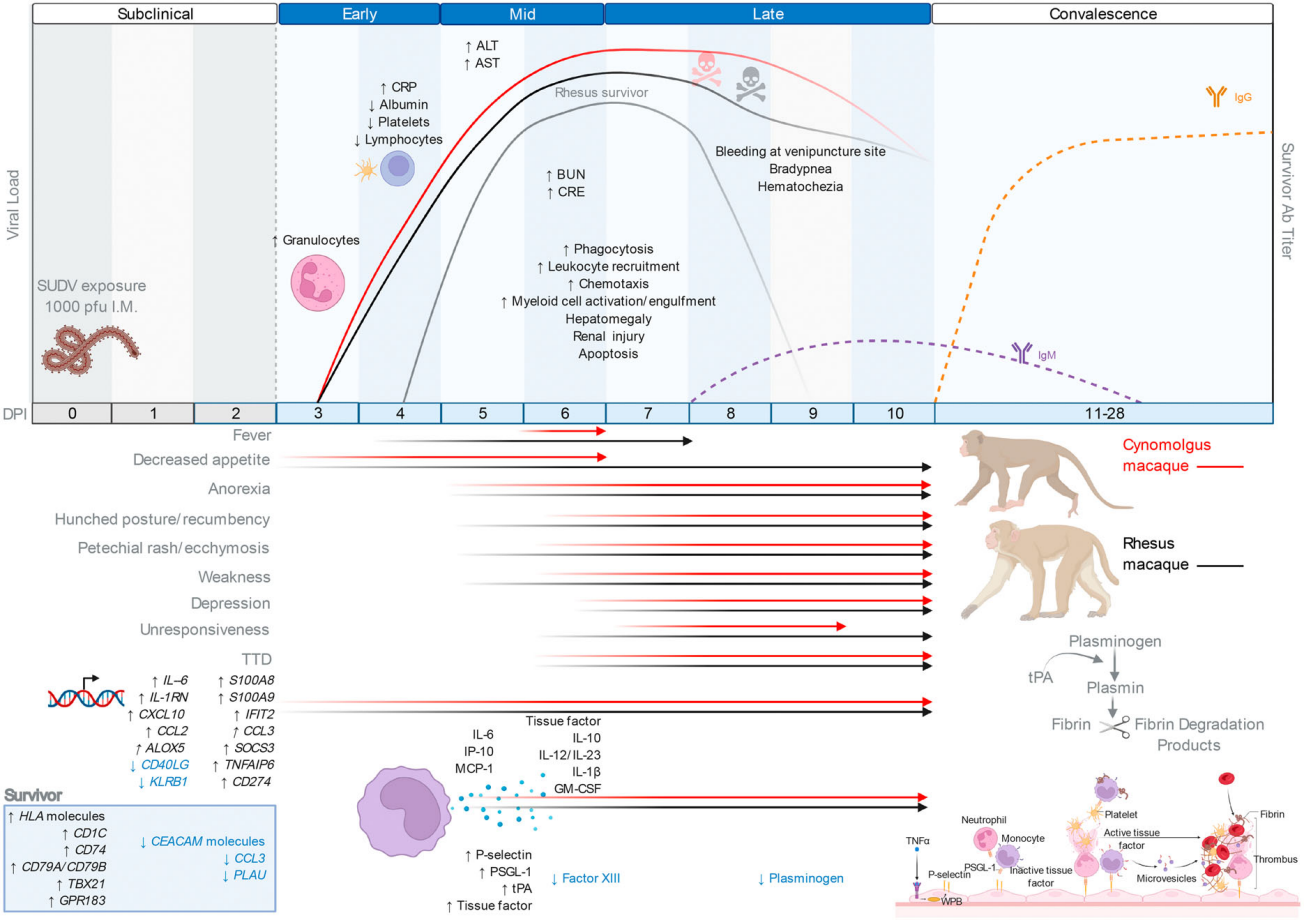
Summary of the most salient features of disease in SUDV-exposed cynomolgus and rhesus macaques. Infected macaques and humans share many disease features including perturbations in liver enzymes (ALT, AST) and kidney function markers (BUN, CRE) along with hemorrhagic manifestations such as a petechial rash and hematochezia. Tissue factor, tPA, and PAI-1 were elevated in infected macaques, which may play a role in disseminated intravascular coagulation, fibrin deposition, and an overactive endothelium, respectively. Transcriptional biomarkers were detectable at the early disease stage followed by shifts in plasma inflammatory and thrombosis mediators at the mid disease stage. The rhesus macaque survivor exhibited only transient shifts in liver and kidney markers, and this subject had a reduced viral load and delayed disease onset in the absence of hemorrhagic manifestations. Survival was also associated with increasing levels of anti-SUDV IgM by the late disease stage followed by increasing levels of anti-SUDV IgG in the convalescent phase. Figure created with BioRender (https://biorender.com/). Abbreviations: SUDV, Sudan virus; I.M, intramuscular; alanine aminotransferase (ALT), aspartate aminotransferase (AST), C-reactive protein (CRP), blood urea nitrogen (BUN), creatine (CRE); DPI, days post infection.

Overall temporal changes in SUDV-infected NHPs are summarized in Figure 7. Specifically, clinical signs, viral load, pathology, transcriptomic findings, and plasma cytokine/chemokine and thrombosis analyte profiles at each stage of disease are highlighted.

## Discussion

The continued re-emergence of ebolaviruses emphasizes the need for animal models that accurately recapitulate human EVD, particularly for less characterized members of the genus such as SUDV, BDBV, TAFV, RESTV, and BOMV. While research focus has been placed on the development of vaccines and treatments against SUDV, there remains a paucity of clinical and preclinical studies to describe the natural progression and pathophysiology of infection with this virus. Here we assessed the pathogenic potential of SUDV infection in CM and RM by i.m. exposure.

Infection with a ∼ 1000 PFU dose of SUDV was 100% lethal in CM and near uniformly lethal in RM (91% mortality rate), with a mean TTD of 7.64 ± 1.72 and 8.3 ± 1.27, respectively. In comparison, the mean TTD for the largest outbreak of SUDV in the Gulu district of Uganda was 8 days after onset of symptoms with a case fatality rate of 53% [34]. The high challenge dose used and i.m. route of infection in this study likely hastened disease progression in NHPs as these factors are known to accelerate the course of EVD [16]. Inoculation via the i.m. route is intended to mimic a worst-case scenario involving an accidental needle stick of either a healthcare worker performing procedures on an infected patient or a laboratory staff member performing procedures on an infected animal. Understanding the disease kinetics of this route of infection is important for identifying the prophylactic window for vaccines and treatments. The clinical pathology and progression of disease were analogous between the two macaque species. These results suggest both CM and RM serve as suitable animal models for SUDV infection. Although we observed earlier detection of fever, rash, and hunched posture in some RM subjects, the disease course appeared slightly faster and more lethal in CM than RM, suggesting the latter model may be more appropriate for treatment studies to evaluate therapeutic effect more sensitively [16].

Immune dysregulation is a hallmark of EVD [1]. Some key features include hypercytokinemia, hyperchemokinemia, and a failed or delayed adaptive response [25]. Infection of NHPs with SUDV led to early and dramatic upregulation of transcripts encoding IP-10, IL-6, MCP-1, pro-inflammatory mediators that were previously reported to correlate with lethality in humans [32,35]. By mid-disease, we detected plasma secretion of these inflammatory proteins. IP-10 and MCP-1 are powerful chemoattractants for monocytes/macrophages and dendritic cells, whereas IL-6 is a molecular control involved in differentiation of monocytes to macrophages [36]. As monocytes, macrophages, and dendritic cells are early and preferred sites of filovirus replication [37], secretion of these cytokines and chemokines likely serves to recruit more cells to the site of infection, thereby promoting dissemination of the virus. This reasoning is in agreement with our hematology and histopathology results.

To identify corelates of natural protection against SUDV infection, we conducted a targeted assessment of the whole blood transcriptome in the single RM survivor. Our results demonstrated the survivor expressed lower expression of granulocyte markers (*CEACAM8*, *CEACAM1*, *CEACAM3*) and higher levels of major histocompatibility complex class II transcripts (e.g. *HLA-DRA*, *HLA-DMB*, *HLA-DMA*, *CD74*). Thus, antigen presentation and formation of an adaptive response is implicated in resistance to SUDV infection, whereas prolonged innate signalling correlates with lethality. This hypothesis is supported by the induction of SUDV-specific IgM and IgG and transcriptional evidence of B- and T-cell activation (*CD79A*, *CD79B*, *TBX21*) in the RM survivor but not fatal subjects. We and others have shown host MHC class II proteins are strikingly downregulated on monocytes following filovirus exposure [38–40] independent of direct infection of these cells [41]. The cytokine milieu, release of immature cells as a consequence of emergency myelopoiesis, or recruitment of myeloid-derived suppressor cells might explain this phenomenon, although the mechanism of MHC class II downregulation is still undetermined [25,41]. Reduced monocyte antigen presentation may contribute to the lack of an adaptive response and ultimate inability to clear the virus.

Our analyses indicate macaques and humans infected with SUDV share many disease features. Coagulopathy and acute liver and kidney injury are significantly associated with severe cases of EVD [1,42,43]. Accordingly, perturbations in liver enzymes (ALT, AST, ALP, GGT) and kidney function markers (BUN, CRE) were prominent findings in fatal macaques along with hemorrhagic manifestations such as petechial rash, epistaxis, and hematochezia. Tissue factor and PAI-1 were also elevated in infected macaques, which may play a role in disseminated intravascular coagulation and an overactive endothelium, respectively [31,44]. Importantly, the single RM survivor exhibited only transient shifts in liver and kidney markers, and this subject had a reduced viral load and delayed disease onset in the absence of hemorrhagic manifestations. While few postmortem examinations in humans with SUDV disease have been carried out, notable findings in two patients resembled those of an acute viral infection with some, but not exclusively, defining EVD features [7]. Some mutual histological findings between SUDV-infected humans and NHPs included focal eosinophilic necrosis of the liver, renal tubular necrosis, massive deposition of fibrin in tissues, and extensive lymphocyte depletion.

In conclusion, this natural history study has expanded our understanding of the pathogenesis of SUDV infection and has enhanced our understanding of immunological factors that mediate natural protection. Our results revealed many similarities between NHP and human SUDV-induced EVD, suggesting the reliability of CM and RM models for medical countermeasure development to fight endemic disease and for biodefense purposes.

## Supplementary Material

Supplemental Material

## Data Availability

All study data are included in the article and/or supplementary material. Transcriptomic fold change and *p*-values are provided as Table S3.
