# Supplementary material for: Natural history of Sudan ebolavirus infection in rhesus and cynomolgus macaques
Source: Emerg Microbes Infect. 2022 Jun 14;11(1):1635–46. doi: 10.1080/22221751.2022.2086072 (PMC9225728; doi:10.1080/22221751.2022.2086072)
Supplement: Supplemental Material [file TEMI_A_2086072_SM2310.zip › Supplemental Information.docx]

## Supporting Information

## Supplemental Methods

## Ethics Statement

Animal studies were conducted in compliance with the Animal Welfare Act and other federal statutes and regulations relating to animal experimentation. Our animal protocols adhered to principles stated in the eighth edition of the *Guide for the Care and Use of Laboratory Animals* established by the National Research Council. The Galveston National Laboratory (GNL) at the University of Texas Medical Branch (UTMB) where this research was conducted is fully accredited by the Association for the Assessment and Accreditation of Laboratory Animal Care International organization and has an approved OLAW Assurance (#A3314-01). Animal studies were performed at biosafety level 4 (BSL-4) biocontainment at UTMB, and protocols were approved by the UTMB Institutional Animal Care and Use Committee (IACUC) and UTMB Institutional Biosafety Committee.

## Challenge virus

The SUDV seed stock (Gulu variant) originates from the serum of a fatal patient (35-year-old male; isolate 200011676) during the 2000-2001 Uganda outbreak (RefSeq #NC_006432). The p2 challenge material was created by passaging the original isolate CDC 808892 (CDC passage 1 of SUDV isolate 200011676) at a MOI of 0.001 onto Vero E6 cells. After 7 days, cell supernatants were harvested as ~ 1 ml aliquots and stored at -80°C. The genetic consensus sequence for this SUDV stock is identical to the parent isolate 808892; the 7U percentage is ~92.33%. Stocks were certified free of endotoxin (< 0.5 EU/mL) and mycoplasma contamination.

## Animal challenge

Fourteen cynomolgus (*Macaca fascicularis*) [19-21] and eleven rhesus (*Macaca mulatta*) [22-25] macaques of Chinese origin (PreLabs, Worldwide Primates) that served as virus positive controls from 17 studies at the GNL were employed for this project. Results from the remaining seven cynomolgus and three rhesus macaques have not been published. All macaques (11 female and 14 male adults) were challenged i.m. in the left quadricep with a 1000 PFU target dose of SUDV variant Gulu. An internal filovirus-specific scoring protocol approved by the UTMB IACUC was implemented to track disease progression in challenged animals. Animals were scored at least twice daily for criteria such as posture/activity level, appetite, behavior, respiration, and the presence of hemorrhagic manifestations. Subjects with a clinical score ≥ 4 (or ≥ 3 for any single parameter) were monitored more frequently (4-8 hours after the afternoon check), and animals that reached a clinical score ≥ 9 were promptly euthanized with a pentobarbital solution. The rhesus survivor was euthanized at the 28 DPI study endpoint.

## Blood collection

Blood was collected by venipuncture into EDTA and serum tubes throughout the course of the study. An aliquot of EDTA-treated whole blood (100 μl) was inactivated with 600 μl of AVL buffer (Qiagen, Hilden, Germany), and RNA was extracted using a Viral RNA mini-kit (Qiagen) according to the manufacturer’s instructions. To isolate plasma and serum, tubes were spun at 2500 rpm for 10 minutes at 4°C. EDTA plasma and serum were stored at -80°C until analysis.

## Hematology and clinical chemistry

Total white blood cell counts, white blood cell differentials, red blood cell counts, platelet counts, hematocrit values, mean cell volumes, mean corpuscular volumes, mean corpuscular hemoglobin concentrations, and total hemoglobin concentrations in EDTA-treated blood were analyzed using a laser-based hematologic analyzer. Serum samples were tested for concentrations of albumin, amylase, alanine aminotransferase (ALT), alkaline phosphatase (ALP), gamma-glutamyltransferase (GGT), aspartate aminotransferase (AST), glucose, cholesterol, total protein, total bilirubin (TBIL), creatine (CRE), blood urea nitrogen (BUN), and C-reactive protein (CRP) using a Piccolo point-of-care analyzer and Biochemistry Panel Plus analyzer discs (Abaxis).

## Viral Load Determination

One-Step Probe RT-qPCR kits (Qiagen) and CFX96 system/software (BioRad) were used to determine viral copies in samples. To detect SUDV RNA, we targeted the *L* gene with primer pairs (forward: 5’-TCA AAT ATT GCA ACC AAT GCT ATG-3’; reverse: 5’-GCA TGT AAC ATT GCG GAA TTA GG-3’; Integrated DNA Technologies) and a 6-carboxyfluorescein (6FAM)-5’- CAT CCA ATC AAA GAC ATT GCG A’-6 carboxytetramethylrhodamine (TAMRA) (Life Technologies) probe. Thermocycler run settings were 50 °C for 10 min, 95 °C for 10 s, and 40 cycles of 95 °C for 10 s and 59 °C for 30 s. Threshold cycle (CT) values representing SUDV *L* genomes were analyzed with CFX Maestro Software, and data are shown as genome equivalents (GEq). To create the GEq standard, RNA from SUDV stocks was extracted, and the number of SUDV *L* genomes was calculated using Avogadro’s number and the molecular weight of the SUDV genome. The limit of detection for this assay is 1000 copies/mL.

Infectious SUDV viral loads were determined using a standard plaque assay. Briefly, increasing 10-fold dilutions of plasma samples were adsorbed to Vero E6 monolayers (ATCC Cat: CRL-1586) in duplicate wells (200 µl), overlaid with 0.8% agarose/2x EMEM, and incubated at 37 °C in 5% CO2. Neutral red stain was added after a 6-day incubation, and plaques were counted after a 24- to 48-hour incubation. The limit of detection for this assay is 25 PFU/mL.

## Transcriptomics

Targeted transcriptomics was performed on macaque whole blood as previously described [26]. NHPV2_Immunology capture and reporter probesets (NanoString Technologies) were hybridized with 5 µl of each RNA sample for ~24 hours at 65°C. The RNA:probeset complexes were subsequently loaded onto an nCounter microfluidics cartridge and assayed using a NanoString nCounter® SPRINT Profiler. Samples with an image binding density greater than 2.0 were re-analyzed with ~2 µl of RNA to meet quality control criteria.

For analysis, nCounter® .RCC files were imported into NanoString nSolver™ 4.0 software. To compensate for varying RNA inputs, an array of housekeeping genes and spiked-in negative and positive controls were used to normalize the raw read counts. Normalized data (fold-change- and p-values) were then exported as a .CSV file. Values were imported into GraphPad Prism version 9.3.1 to produce the transcript heatmap. The data was then analyzed with NanoString nSolver™ Advanced Analysis 2.0 package to generate principal component (PC) figures, biomarker trend plots, and the volcano plot. Functional enrichment of normalized counts was performed at the time of challenge, and early, mid, and late disease with Ingenuity Pathway Analysis (Qiagen). Z-scores were imported into GraphPad Prism version 9.3.1 to produce the canonical signaling, upstream regulator, tox functions, and diseases and functions heatmaps. Immune cell profiling was accomplished via CIBERSORT web-based deconvolution software [27] using the LM22 signature matrix file. The 6-way Venn diagram was created with InteractiVenn [28].

## Bead-based multiplex assays

Plasma inflammatory mediators, thrombosis markers, and fibrinolysis analytes were measured by flow cytometry using Biolegend LegendPlex™ assays and a FACS Canto-II cytometer (Becton Dickson). Samples were processed in duplicate for each Nonhuman Primate Inflammation 13-plex (1:4 dilution), Human Thrombosis (1:100), and Human Fibrinolysis (1:40,000) panel according to the manufacturer’s instructions. To ensure consistency among plates, standards were mixed in batch and aliquoted across all plates. Optional wash steps were incorporated to reduce background. Fold change calculations were plotted using the package Pheatmap v1.0.12 in R. Results of fold change calculations and analysis of variance (ANOVA) with Tukey post-hoc test were calculated and plotted using the ggplot2 (v3.3.5) [28], ggbreak (v0.0.8) [29], viridis (v0.6.2) [30], and rstatix (v0.7.0) packages.

***Histopathology and immunohistochemistry***

Tissue samples of all major organs were harvested in 10% neutral buffered formalin for histopathologic and immunohistochemical (IHC) examination. Tissue sections were deparaffinized and rehydrated through a series of xylene and graded ethanol washes followed by a distilled water rinse. Subsequently, slides went through heat antigen retrieval in a steamer at 95°C for 20 minutes in Sigma Citrate Buffer, pH6.0, 10x (Sigma Aldrich, St. Louis, MO). To block endogenous peroxidase activity, slides were treated with 3% hydrogen peroxide and rinsed in distilled water. The tissue sections were processed for IHC using the Thermo Autostainer 360 (ThermoFisher, Kalamazoo, MI). To block endogenous biotin reactivity, sequential 15-minute incubations with avidin D and biotin solutions (Vector Laboratories, Burlingame, CA #SP-2001) were performed. Both primary antibodies were combined before application, explicitly, virus specific anti-Ebola Sudan VP40 (IBT #0302-001) immunoreactivity was detected using an anti-Ebola Sudan VP40 primary antibody at a 1:4000 dilution and anti-CD68 was detected using an Anti-CD68 primary antibody at a dilution of 1:250 (Abcam# ab283316) for 60 min. After incubation with the primary antibody mix, biotinylated goat anti-mouse IgG (Vector Laboratories, Burlingame, CA #BA-9200) was applied at 1:200 for 30 min followed by Vector Horseradish Peroxidase Streptavidin, R.T.U (Vector Laboratories #SA-5704) for 30 min. The CD68 was then developed with Dako DAB chromogen (Dako, Carpenteria, CA #K3468) for 5 min. After developing the first marker of interest the secondary antibody added was biotinylated goat anti-rabbit IgG (Vector Laboratories, Burlingame, CA #BA-1000) at 1:200 for 30 min followed by Vector Streptavidin Alkaline Phosphatase at a dilution of 1:200 for 20 minutes (Vector Laboratories #SA-5100). The SEBOV VP40 was then developed with Bio-Red (Biopath Laboratories, Oklahoma City, OK #BP-100-FR) for 7 min and counterstained with hematoxylin for 35 seconds. Specific anti-Fibrin was detected using an anti-Fibrin primary antibody at a 1:3200 dilution for 60 min. Secondary antibody used was biotinylated goat anti-mouse IgG (Vector Laboratories, Burlingame, CA #BA-9200) at 1:200 for 30 min followed by Vector Streptavidin Alkaline Phosphatase at a dilution of 1:200 for 20 minutes (Vector Laboratories #SA-5100). Slides were developed with Bio-Red (Biopath Laboratories, Oklahoma City, OK #BP-100-FR) for 7 min and counterstained with hematoxylin for 45 seconds. Slides were reviewed by a board-certified veterinary pathologist.

## Statistical analysis

Statistical comparisons of rhesus versus cynomolgus macaque cohorts including survival, viral loads, hematology data, serum biochemistry values, and multiplex assay findings were carried out in GraphPad Prism version 9.3.1 (GraphPad, Software, Inc., La Jolla, CA) using multiple Mann-Whitney tests with the two-stage step-up method (Benjamini, Krieger, and Yekutieli) for multiple comparisons. No significant difference between cynomolgus and rhesus macaque groups was detected for any measured parameter. A multiple hypothesis Benjamini-Hochberg false discovery rate (FDR) corrected p-value less than 0.05 was deemed significant for transcriptional analyses, unless otherwise stated. An analysis of variance (ANOVA) with Tukey post-hoc test was used to determine plasma marker significance.

**Table S1. Clinical description and outcome of cynomolgus macaques following SUDV challenge**

| **NHP** | **Sex** | **Clinical illness** | **Clinical pathology** |
| --- | --- | --- | --- |
| CYNO-1 | F | Decreased appetite (d5); depression (d5, 6); anorexia (d6); weakness (d6); hunched posture (d6); recumbency (d6); petechial rash (d6); hemorrhage from venipuncture site (d6). Subject euthanized (d6). | Thrombocytopenia (d3, 6); monocytosis (d3); granulocytopenia (d6); hypoalbuminemia (d6); BUN ↑ (d6); CRE ↑ (d6); ALT ↑↑ (d6); ALP ↑↑ (d6); GGT ↑ (d6); CRP ↑↑↑↑ (d6). |
| CYNO-2 | M | Fever (d6); anorexia (d6, 7); depression (d6, 7); weakness (d7); petechial rash (d6, 7). Subject succumbed to disease(d8). | Thrombocytopenia (d6); monocytopenia (d3, 6); granulocytosis (d3, 6); hypoalbuminemia (d6); hypoamylasemia (d6); AST ↑↑ (d6); CRP ↑↑↑↑ (d6). |
| CYNO-3 | M | Fever (d6); depression (d6-10); anorexia (d6-10); hunched posture (d6-10); depression (d6-10); petechial rash (d6-10); weakness (7-10); facial flushing (d7, 8); ecchymosis (d10); epistaxis (d10); recumbency (d10). Subject euthanized (d10). | Thrombocytopenia (d6, 10); lymphocytopenia (d10); monocytopenia (d10); granulocytosis (d6, 10); hypoalbuminemia (d6, 10); hypoamylasemia (d6); BUN ↑↑↑ (d10); CRE ↑↑ (d10); ALT ↑ (d10); AST ↑ (d6), ↑↑↑ (d10); GGT ↑↑ (d10); ↑↑↑↑ (d6, 10). |
| CYNO-4 | M | Decreased appetite (d5); anorexia (d6); depression (d6); weakness (d6); hunched posture (d6); petechial rash (d6); ecchymosis (d6). Subject succumbed to disease (d6). | Lymphocytopenia (d6); thrombocytopenia (d6); monocytopenia (d6); monocytosis (d3); granulocytosis (d3, 6); hypoalbuminemia (d6); hypoamylasemia (d6); BUN ↑ (d6); CRE ↑ (d6); ALT ↑↑↑ (d6); AST ↑ (d6); ALP ↑ (d6); CRP ↑ (d3), ↑↑↑↑ (d6). |
| CYNO-5 | F | Decreased appetite (d5); anorexia (d6); depression (d6); hunched posture (d6); petechial rash (d6); weakness (d6); recumbency (d6). Subject euthanized (d6). | Thrombocytopenia (d6); monocytopenia (d6); monocytosis (d3); granulocytosis (d3); hypoalbuminemia (d6); hypoamylasemia (d6); BUN ↑ (d6); CRE ↑ (d6); ALT ↑↑↑ (d6); AST ↑↑↑↑ (d6); ALP ↑ (d6); GGT ↑ (d6); CRP ↑↑↑↑ (d6). |
| CYNO-6 | M | Fever (d6); anorexia (d6-8); depression (d6-8); hunched posture (d6-8); petechial rash (d6-8); weakness (d8); epistaxis (d8). Subject euthanized (d8). | Lymphocytopenia (d6, 8); thrombocytopenia (d6, 8); monocytopenia (d6, 8); granulocytosis (d6, 8); hypoglycemia (d6); hypoalbuminemia (d6, 8); hypoamylasemia (d6); hypocalcemia (d8); BUN ↑↑ (d8); CRE ↑↑↑ (d8); ALT ↑ (d8); AST ↑ (d6), ↑↑↑↑ (d8); ALP ↑ (d6, 8); GGT ↑ (d8); CRP ↑↑↑↑ (d6, 8). |
| CYNO-7 | F | Decreased appetite (d1, 5); anorexia (d6-8); petechial rash (d6-8); depression (d8); hunched posture (d8); weakness (d8); recumbency (d8); bradypnea (d8); unresponsiveness (d8). Subject euthanized (d8). | Lymphocytopenia (d6, 8); thrombocytopenia (d6, 8); monocytopenia (d6); granulocytosis (d8); hypoalbuminemia (d6, 8); hypoamylasemia (d6); BUN ↑ (d8); CRE ↑ (d8); AST ↑ (d6), ↑↑ (d8); ALP ↑ (d6, 8); CRP ↑↑↑ (d6, 8). |
| CYNO-8 | F | Decreased appetite (d0-4); anorexia (d5-9); fever (d6); depression (d8, 9); hunched posture (d8); weakness (d9); recumbency (d9); petechial rash (d9); hematochezia (d9); ascites (d9). Subject euthanized (d9). | Lymphocytopenia (d6, 9); thrombocytopenia (d6, 9); monocytopenia (d9); granulocytosis (d3, 6, 9); hypoglycemia (d9); hypoalbuminemia (d6, 9); hypoamylasemia (d6); ALP ↑ (d6), ↑↑↑ (d9); CRP ↑ (d3), ↑↑↑↑ (d6, 9). |
| CYNO-9 | M | Decreased appetite (d1, 2, 4); anorexia (d5-7); depression (d6, 7); hunched posture (d6); petechial rash (d6, 7); weakness (d7); recumbency (d7); bradypnea (d7). Subject euthanized (d7). | Thrombocytopenia (d6, 7); monocytopenia (d6); granulocytopenia (d6); granulocytosis (d3); hypoglycemia (d7); hypoalbuminemia (d6, 7); hypoamylasemia (d6); BUN ↑ (d6, 7); CRE ↑ (d6, 7); ALT ↑↑ (6, 7); AST ↑↑↑ (d6), ↑↑↑↑ (d7); ALP ↑(d6); CRP ↑↑↑↑ (d6, 7). |
| CYNO-10 | F | Decreased appetite (d1, 2, 5); anorexia (d6, 7); petechial rash (d6, 7); hunched posture (d6, 7); depression (d7); weakness (d7); recumbency (d7); unresponsiveness (d7). Subject euthanized (d7). | Lymphocytopenia (d6); thrombocytopenia (d6, 7); monocytosis (d3); granulocytosis (d3, 6, 9); hypoglycemia (d7); hypocalcemia (d7); hypoalbuminemia (d7); hypoamylasemia (d6); BUN ↑ (d7); CRE ↑↑ (d7); ALT ↑↑↑ (d7); AST ↑ (d6), ↑↑↑↑ (d7); ALP ↑ (d6), ↑↑ (d7); GGT ↑ (d7); CRP ↑↑↑ (d6, 7). |
| CYNO-11 | F | Decreased appetite (d0-5); anorexia (d6, 7); hunched posture (d6); facial flushing (d6). Subject succumbed to disease (d7). | Lymphocytopenia (d4); thrombocytopenia (d7); monocytopenia (d7); granulocytosis (d4, 7); hypoglycemia (d7); hypoalbuminemia (d7); hypoamylasemia (d4, 7); BUN ↑ (d7); CRE ↑ (d7); ALT ↑↑ (d7); AST ↑↑↑↑ (d7); ALP ↑↑(d7); GGT ↑ (d7); CRP ↑↑↑↑ (d4, 7). |
| CYNO-12 | M | Decreased appetite (d5); anorexia (d6-8); fever (d7); depression (d7, 8); hunched posture (d7, 8); petechial rash (d7, 8); weakness (d8); recumbency (d8); dyspnea (d8); unresponsiveness (d8). Subject euthanized (d8). | Monocytosis (d4, 8); granulocytosis (d4, 7, 8); hypoglycemia (d7); hypocalcemia (d8); hypoalbuminemia (d7, 8); hypoamylasemia (d4); BUN ↑ (d7), ↑↑ (d8); CRE ↑↑ (d7, 8); ALT ↑ (d7, 8); AST ↑↑↑↑ (d7, 8); ALP ↑ (d7, 8); GGT ↑ (d7, 8); CRP ↑↑↑ (d4), ↑↑↑↑ (d7, 8). |
| CYNO-13 | M | Decreased appetite (d5); anorexia (d6-9); petechial rash (7-9); depression (d9); hunched posture (d9); diarrhea (d9); hyphema (d9); weakness (d9); recumbency (d9); unresponsiveness (d9); hematochezia (d9). Subject euthanized (d9). | Lymphocytopenia (d7, 9); thrombocytopenia (d7, 9); basopenia (d7, 9); monocytosis (d4); neutrophilia (d4, 9); eosinophilia (d4, 9); hypoglycemia (d9); hypocalcemia (d9); hypoalbuminemia (d7, 9); BUN ↑ (d7), ↑↑ (d9); CRE ↑ (d7), ↑↑↑ (d9); ALT ↑ (d7), ↑↑↑↑ (d9); AST ↑↑↑ (d7), ↑↑↑↑ (d9); ALP ↑ (d7, 9); GGT ↑ (d9); CRP ↑↑↑↑ (d4, 7, 9). |
| CYNO-14 | F | Decreased appetite (d6); anorexia (d7, 8); depression (d7, 8); hunched posture (d7); weakness (d8); petechial rash (d8); recumbency (d8); unresponsiveness (d8). Subject euthanized (d8). | Lymphocytopenia (d7, 8); thrombocytopenia (d7, 8); eosinophilia (d7, 8); hypoalbuminemia (d8); hypoamylasemia (d7); BUN ↑ (d8); CRE ↑ (d8); ALT ↑ (d8); AST ↑↑ (d7), ↑↑↑ (d8); ALP ↑↑ (d7), ↑↑↑↑ (d8); GGT ↑ (d7, 8); CRP ↑ (d4), ↑↑↑↑ (d7, 8). |
|  |  |  |  |

Days after SUDV challenge are in parentheses. All reported findings are in comparison to baseline (d0) values. Decreased appetite is defined as some food but not all food consumed from the previous day. Anorexia is defined as no food consumed from the previous day. Fever is defined as a temperature more than 2.5 °F over baseline, or at least 1.5 °F over baseline and ≥ 103.5 °F. Hypothermia is defined as a temperature ≤3.5°F below baseline. Lymphocytopenia, thrombocytopenia, monocytopenia, neutropenia, eosinopenia, and basopenia are defined by a ≥35% drop in numbers of lymphocytes, platelets, monocytes, neutrophils, eosinophils, or basophils, respectively. Lymphocytosis, thrombocytosis, monocytosis, neutrophilia, eosinophilia, and basophilia are defined by a 100% or greater increase in numbers of lymphocytes, platelets, monocytes, neutrophils, eosinophils, or basophils, respectively. Granulocytopenia and granulocytosis are defined by a ≥35% decrease or 100% increase in some or all subpopulations of granulocytes (neutrophils, eosinophils, and basophils), respectively. Hyperglycemia is defined as a 100% or greater increase in levels of glucose. Hypoglycemia is defined by a ≥25% decrease in levels of glucose. Anemia is defined as a concurrent ≥25% decrease in erythrocyte count, Hct, and Hgb. Hypoalbuminemia is defined by a ≥25% decrease in levels of albumin. Hypoproteinemia is defined by a ≥25% decrease in levels of total protein. Hypoamylasemia is defined by a ≥25% decrease in levels of serum amylase. Hypocalcemia is defined by a ≥25% decrease in levels of serum calcium. Increases in ALT, AST, ALP, CRE, CRP, Hct, and Hgb were graded on the following scale: ↑ = 1-5 fold, ↑↑ = >5-10 fold, ↑↑↑ = >10-20 fold, ↑↑↑↑ = >20-fold, ↓ = ≥50% decrease. (BUN) blood urea nitrogen, (ALT) alanine aminotransferase, (AST) aspartate aminotransferase, (ALP) alkaline phosphatase, (CRE) Creatinine, (CRP) C-reactive protein, (Hct) hematocrit, (Hgb) hemoglobin, (i.m.) intramuscular.

**Table S2. Clinical description and outcome of rhesus macaques following SUDV challenge.**

| **NHP** | **Sex** | **Clinical illness** | **Clinical pathology** |
| --- | --- | --- | --- |
| RHES-1 | F | Anorexia (d5-10); depression (d5-10); hunched posture (d7-10); petechial rash (d7-10); weakness (d7-10); facial flushing (d8-10); conjunctivitis (d9); ecchymosis (d10); dyspnea (d10); hemorrhage from venipuncture site (d10); recumbency (d10); unresponsiveness (d10); epistaxis (d10). Subject euthanized (d10). | Lymphocytopenia (d6, 10); thrombocytopenia (d6, 10); monocytosis (d3); granulocytosis (d3, 6, 10); hypoglycemia (d10); hypoalbuminemia (d6, 10); hypoamylasemia (d6, 10); BUN ↑↑ (d10); CRE ↑ (d10); ALT ↑ (d10); AST ↑↑↑ (d10); ALP ↑ (d6), ↑↑ (d10); GGT ↑ (d10); CRP ↑↑↑↑ (d6, 10). |
| RHES-2 | M | Decreased appetite (d4); anorexia (d5-8); depression (d5-8); hunched posture (d5-8); hematochezia (d5, 8); facial flushing (d6, 7); petechial rash (d6-8); weakness (d7, 8); recumbency (d8); dyspnea (d8); unresponsiveness (d8); ecchymosis (d8). Subject euthanized (d8). | Lymphocytopenia (d6); thrombocytopenia (d6, 8); monocytopenia (d6, 8); granulocytosis (d3, 6, 8); hypoalbuminemia (d6, 8); hypoamylasemia (d6); BUN ↑ (d6), ↑↑↑ (d8); CRE ↑ (d6), ↑↑ (d8); ALT ↑ (d6), ↑↑ (d8); AST ↑↑ (d6), ↑↑↑↑ (d8); ALP ↑ (d6); GGT ↑ (d8); CRP ↑ (d3), ↑↑↑↑ (d6, 8). |
| RHES-3 | M | Decreased appetite (d5); anorexia (d6-8); depression (d8); hunched posture (d8); petechial rash (d8). Subject succumbed to disease (d9). | Lymphocytopenia (d6); monocytosis (d3); granulocytosis (d6); hypoamylasemia (d6); AST ↑ (d6); CRP ↑↑↑↑ (d6). |
| RHES-4 | M | Fever (d4-6); decreased appetite (d5); anorexia (d6-9); depression (d6-9); hunched posture (d6-9); petechial rash (d6-9); recumbency (d6, 7, 9); facial flushing (d7); weakness (d9); unresponsiveness (d9); epistaxis (d9); hematochezia (d9); hemorrhage from venipuncture site (d9); dehydration (d9). Subject euthanized (d9). | Lymphocytopenia (d9); thrombocytopenia (d7, 0); monocytopenia (d7, 9); monocytosis (d3); granulocytosis (d4, 9); hypoglycemia (d7, 9); hypoalbuminemia (d7, 9); hypoamylasemia (d7, 9); BUN ↑ (d7, 9); CRE ↑ (d7); ALT ↑↑ (d7, 9); AST ↑↑↑↑ (d7, 9); GGT ↑ (d9); CRP ↑↑ (d4), ↑↑↑ (d7), ↑ (d9). |
| RHES-5 | F | Fever (d5); decreased appetite (d5); anorexia (6-9); depression (d6-9); hunched posture (d6-9); petechial rash (d8, 9); facial flushing (d8); abdominal distension (d8, 9); weakness (d9); recumbency (d9); unresponsiveness (d9); ecchymosis (d9); epistaxis (d9). Subject euthanized (d9). | Lymphocytopenia (d5); thrombocytopenia (d8, 9); monocytopenia (d5, 8, 9); granulocytosis (d5, 9); hypoglycemia (d9); hypoalbuminemia (d8, 9); hypoamylasemia (d5, 8, 9); ALT ↑ (d8, 9); AST ↑↑ (d8, 9); GGT ↑ (d8, 9); CRP ↑↑↑↑ (d5), ↑ (d8, 9). |
| RHES-6 | M | Fever (d4, 5); anorexia (d5-8); conjunctivitis (d5); petechial rash (d5-8); ecchymosis (d8); weakness (d8); recumbency (d8); unresponsiveness (d8). Subject euthanized (d8). | Lymphocytopenia (d4, 5); thrombocytopenia (d5, 8); monocytosis (d5); granulocytosis (d4, 5, 8); hypoglycemia (d8); hypalbuminemia (d5, 8); hypoamylasemia (d4, 5, 8); BUN ↑ (d8); CRE ↑ (d8); ALT ↑ (d8); AST ↑ (d5), ↑↑↑↑ (d8); GGT ↑ (d8); CRP ↑↑↑↑ (d8). |
| RHES-7 | M | Fever (d5); anorexia (d6-10); decreased appetite (d11-18). Subject survived to study endpoint (d28). | Lymphocytopenia (d8, 11); thrombocytopenia (d8, 11); monocytopenia (d11); monocytosis (d3, 5, 14); granulocytosis (d3, 5, 8, 11, 14, 21); hypoglycemia (d11); hypoalbuminemia (d8, 11, 14, 21); hypoamylasemia (d5, 8, 11, 14); AST ↑↑ (d11), ↑ (d14); CRP ↑↑↑ (d5, 8), ↑↑ (d11). |
| RHES-8 | M | Fever (d4, 7); decreased appetite (d6, 7, 10); anorexia (d8, 9); depression (d9, 10); hunched posture (d9, 10); weakness (d10); recumbency (d10); hemorrhage from i.m. injection site (d10); petechial rash (d10). Subject euthanized (d10). | Lymphocytopenia (d4, 7, 10); thrombocytopenia (d7, 10); monocytopenia (d4, 10); basopenia (d7, 10); monocytosis (d7); neutrophilia (d4, 7, 10); eosinophilia (d4); basophilia (d4); hypoglycemia (d10); hypoalbuminemia (d7, 10); hypoamylasemia (d4, 7, 10); ALT ↑ (d7); AST ↑↑ (d7), ↑↑↑ (d10); ALP ↑ (d7, 10); GGT ↑ (d10); CRP ↑↑↑↑ (d4, 7, 10). |
| RHES-9 | F | Fever (d5); decreased appetite (d1, 2, 4); anorexia (d5-7); petechial rash (d6, 7); depression (d7); weakness (d7); recumbency (d7); unresponsiveness (d7). Subject euthanized (d7). | Lymphocytopenia (d5); thrombocytopenia (d7); monocytosis (d5, 7); neutrophilia (d5, 7); eosinophilia (d5); basophilia (d5); hypoalbuminemia (d7); hypoamylasemia (d5, 7); CRE ↑ (d7); ALT ↑ (d7); AST ↑ (d5), ↑↑↑ (d7); ALP ↑ (d5), ↑↑↑ (d7); GGT ↑ (d7); CRP ↑↑↑↑ (d5, 7) |
| RHES-10 | F | Decreased appetite (d5); hunched posture (d5, 6); weakness (d5, 6); anorexia (d6); depression (d6); petechial rash (d6); ecchymosis (d6); unresponsiveness (d6). Subject euthanized (d6). | Lymphocytopenia (d6); thrombocytopenia (d6); monocytosis (d6); neutrophilia (d6); eosinophilia (d6); hypocalcemia (d6); hypoalbuminemia (d6); hypoamylasemia (d6); BUN ↑ (d6); CRE ↑↑ (d6); ALT ↑↑ (d6); AST ↑↑↑↑ (d6); ALP ↑ (d6); GGT ↑↑ (d6); CRP ↑↑↑↑ (d6). |
| RHES-11 | F | Decreased appetite (d0-2, 4); anorexia (d5-7); weakness (d5-7); hunched posture (d5, 6); petechial rash (d6, 7); depression (d7); recumbency (d7); unresponsiveness (d7). Subject euthanized (d7). | Lymphocytopenia (d5, 7); thrombocytopenia (d7); monocytopenia (d7); neutropenia (d5, 7); eosinopenia (d7); basopenia (d7); monocytosis (d5); hypoalbuminemia (d7); hypoamylasemia (d5, 7); BUN ↑ (d7); CRE ↑ (d7); ALT ↑↑ (d7); AST ↑↑↑↑ (d7); ALP ↑ (d7); GGT ↑ (d7); CRP ↑↑↑↑ (d5, 7). |
|  |  |  |  |

Days after SUDV challenge are in parentheses. All reported findings are in comparison to baseline (d0) values. Decreased appetite is defined as some food but not all food consumed from the previous day. Anorexia is defined as no food consumed from the previous day. Fever is defined as a temperature more than 2.5 °F over baseline, or at least 1.5 °F over baseline and ≥ 103.5 °F. Hypothermia is defined as a temperature ≤3.5°F below baseline. Lymphocytopenia, thrombocytopenia, monocytopenia, neutropenia, eosinopenia, and basopenia are defined by a ≥35% drop in numbers of lymphocytes, platelets, monocytes, neutrophils, eosinophils, or basophils, respectively. Lymphocytosis, thrombocytosis, monocytosis, neutrophilia, eosinophilia, and basophilia are defined by a 100% or greater increase in numbers of lymphocytes, platelets, monocytes, neutrophils, eosinophils, or basophils, respectively. Granulocytopenia and granulocytosis are defined by a ≥35% decrease or 100% increase in some or all subpopulations of granulocytes (neutrophils, eosinophils, and basophils), respectively. Hyperglycemia is defined as a 100% or greater increase in levels of glucose. Hypoglycemia is defined by a ≥25% decrease in levels of glucose. Anemia is defined as a concurrent ≥25% decrease in erythrocyte count, Hct, and Hgb. Hypoalbuminemia is defined by a ≥25% decrease in levels of albumin. Hypoproteinemia is defined by a ≥25% decrease in levels of total protein. Hypoamylasemia is defined by a ≥25% decrease in levels of serum amylase. Hypocalcemia is defined by a ≥25% decrease in levels of serum calcium. Increases in ALT, AST, ALP, CRE, CRP, Hct, and Hgb were graded on the following scale: ↑ = 1-5 fold, ↑↑ = >5-10 fold, ↑↑↑ = >10-20 fold, ↑↑↑↑ = >20-fold, ↓ = ≥50% decrease. (BUN) blood urea nitrogen, (ALT) alanine aminotransferase, (AST) aspartate aminotransferase, (ALP) alkaline phosphatase, (CRE) Creatinine, (CRP) C-reactive protein, (Hct) hematocrit, (Hgb) hemoglobin.
